# Strengthening the competitiveness of EU *in vitro* biotechnologies

**DOI:** 10.1016/j.tibtech.2025.07.019

**Published:** 2026-02

**Authors:** Milena Mennecozzi, Monica Piergiovanni, Lucia Selfa Aspiroz, Maurice Whelan

**Affiliations:** 1 European Commission, Joint Research Centre (JRC), Ispra, Italy

**Keywords:** *in vitro*, biotechnology, EU competitiveness, market analysis, industrial ecosystem

## Abstract

The EU has been a leader in research and development of *in vitro* biotechnology (IVB), such as human 3D cell and tissue models used in disease research and drug development. However, it is struggling to convert scientific discoveries into business creation and competitiveness within a rapidly growing international market.

## EU biotechnology

The ‘Boosting Biotechnology and Biomanufacturing in the EU’ Communicationi highlights the crucial role of biotechnology for enhancing the competitiveness and modernisation of European industries. Biotechnology can address pressing societal and environmental challenges, including climate change, sustainable use of natural resources, food supply, and human health. The OECD (Organisation for Economic Cooperation and Development) defines biotechnology as the application of science and technology to living organisms to modify them for the purpose of generating knowledge, products, and servicesii.

The Draghi Competitiveness Reportiii emphasises the importance of investing in research and development, identifying biotechnology as a key area. The EU needs to stay at the forefront of technological advancements to remain competitive in global markets. This priority is reflected in various sector-related policy initiatives, such as the Pharmaceutical Strategy for Europeiv, the EU Chemicals Strategy for Sustainabilityv, and the European Industrial Strategyvi. Furthermore, the concept of Open Strategic Autonomyvii, which promotes European autonomy and self-sufficiency in critical technologies, and the Strategic Transformation Enablement Programme (STEP) platformviii both include biotechnology. The EU valorisation policyix drives the commercialisation of research outcomes by bringing new products and services to market. The European Commission’s Roadmap for phasing out animal testing for chemicals assessmentx highlights biotechnologies as key enablers for the development of alternative methods and approaches, while the Commission’s recently published Life Science Strategyxi outlines initiatives that are expected to further steer the development, access to market, and uptake of biotechnology across various sectors, including research, medicines, agriculture, and industry.

## IVB definition and market segmentation

IVB is a rapidly growing sector that combines advanced micro-engineered technologies with biological materials to produce a range of novel tools used in life science research and product development. Common IVB materials include patient-derived primary cells, tissues, organoids, genetically modified cells, induced pluripotent stem cells (iPSCs), bacteria, viruses, and biomolecules (e.g., proteins, DNA, RNA, mRNA). Essential consumables include culture media, plates, scaffolds, and other laboratory reagents necessary for maintaining and manipulating biological samples. Devices such as microfluidic chips and 3D bioprinters enable the development of advanced models that mimic biological functions such as breathing or heartbeat. Combinations of biological materials with these devices are known as microphysiological systems (MPS) or organ-on-chip (OoC) technology. Additionally, high-throughput screening (HTS) instruments and software, including robotic workstations and liquid handling systems, facilitate large-scale processing and analysis of biological samples, significantly accelerating research and development workflows.

IVB is used in a variety of fields for both research and industrial purposes. Biomedical research uses IVB to understand how living organisms are formed and how they function. For example, IVB models based on iPSCs recapitulate features of physiology and pathologies, such as respiratory tract and neurodegenerative disorders [1,2], providing insights into disease mechanisms. Pharmaceutical companies use IVB in drug discovery and development to assess the efficacy and safety of novel medicines. For instance, data generated using an OoC have been successfully used in drug repurposing for a rare disease [3].

The food industry also relies on IVB to assess the safety of chemicals used in food, including additives, residues from animal medicines, contaminants, and natural toxins. The Developmental Neurotoxicity (DNT) *in vitro* battery, which employs high content imaging measurements of complex 3D cellular models, is currently used for the safety assessment of pesticides [4]. Beyond pharmaceuticals and food safety, IVB is increasingly used in (toxicological) risk assessment across other industry sectors, including consumer care, medical devices, and industrial chemicals. For instance, OECD Test Guideline 431 assesses the potential risk for skin corrosion using reconstructed (engineered) human epidermis [5].

## IVB market analysis

The global market for cell-based technologies, including 3D models, covers the sales of biological materials, devices and consumables, instruments and software, and services. In 2023, this market was worth US$19.6 billion and is projected to reach US$30.7 billion by 2028. Within this sector, Europe accounted for the second largest market share at 28.2%, with an anticipated compound annual growth rate (CAGR) of 9% for 2023–2028 [6,7]. Similarly, the HTS market was worth US$25.7 billion in 2023 and is projected to reach US$44.5 billion by 2028. Europe again holds the second largest share (26.8%) with an estimated CAGR of 10.8% for 2023–2028 [8]. North America dominated both markets (41.7% for the cell-based and 42% for the HTS), while Asia ranked third (22.2% and 22.7%, respectively) but it is projected to grow with the highest CAGR (10.3% and 13%, respectively) [6., 7., 8.].

**Figure 1:**
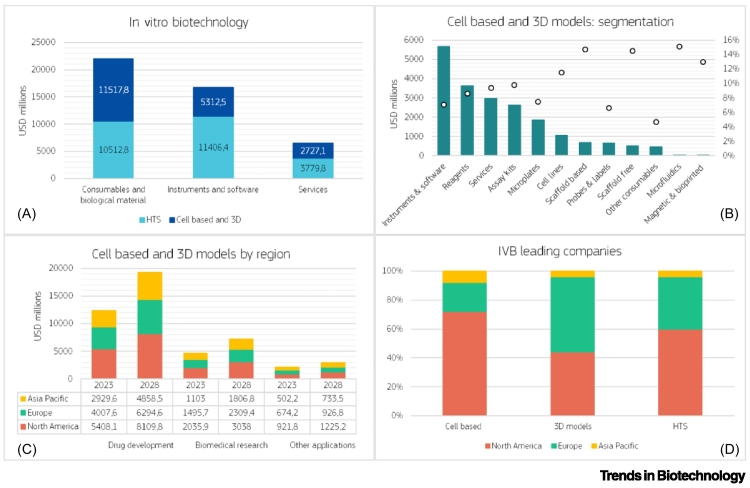
*In vitro biotechnology (*IVB) market analysis. (A) Worldwide market dimension. (B) Details on the main products in the cell-based and 3D model category. (C) EU competitiveness compared with the USA and China. (D) Distribution of key industrial players by region. All data refer to 2023 and graphs are based on the Markets and Markets reports [6., 7., 8.]. Abbreviation: HTS, high-throughput screening.

Overall, the IVB market in 2023 exceeded US$56 billion. Nearly 50% of this market is represented by consumables, 34% by instruments and software, and 16% by services (Figure 1). IVB adoption in biomedical research is growing due to its increasing capability to replicate complex biology (Figure 1C). Leading IVB companies include Danaher Corp., ThermoFischer Scientific, Merck, Corning, Lonza, Tecan Trading Ag, and Perkin-Elmer. Most of them have their headquarters in North America, while many European companies offer more advanced and innovative 3D solutions (Figure 1D).

## IVB value chain and SWOT (strengths, weaknesses, opportunities, threats) analysis

**Figure 2:**
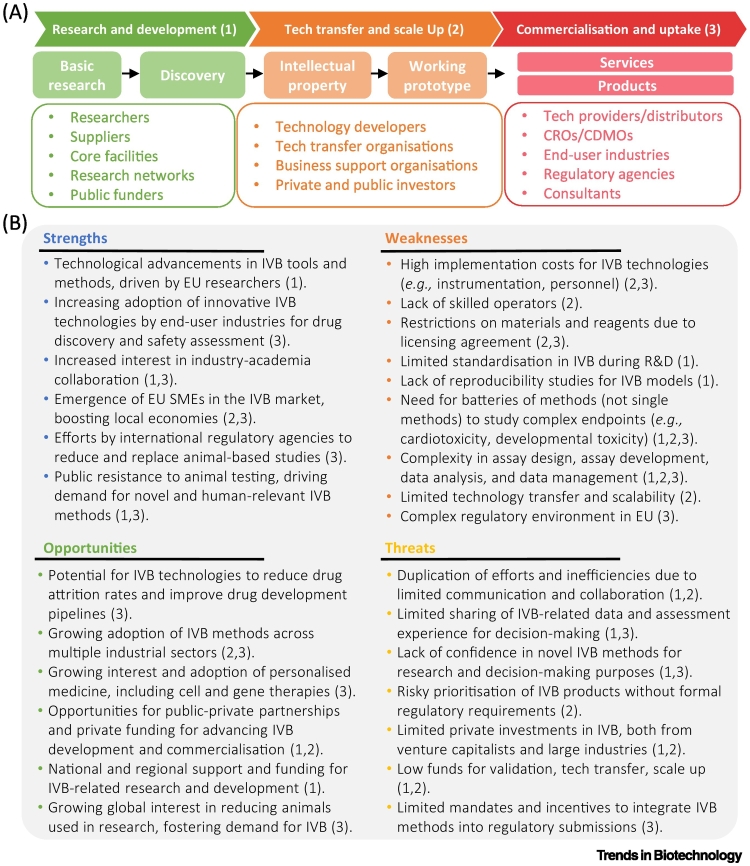
*In vitro* biotechnology (IVB) value chain and SWOT (strengths, weaknesses, opportunities, threats) analysis. Representation of the IVB ecosystem value chain and the main stakeholders involved in each step of the process. (A) The three main stages of the value chain (1), (2), and (3) are shown. (B) SWOT analysis for developing IVB products and services. Each element of the SWOT analysis associated to one or multiple stages of the numbered value chain: research and development (1), transfer and scale-up (2), commercialisation and uptake (3). Abbreviations: CDMOs, contract development and manufacturing organisations; CROs, contract research organisations; Tech, technology.

The IVB ecosystem is a dynamic network driving innovation and scientific advancement (Figure 2A). Researchers transform their ideas into novel technologies supported by core facilities, suppliers, and research consortia that facilitate knowledge exchange, partnerships, and capacity building. Technology transfer organisations (e.g., tech transfer offices, technology support centres, science and technology parks, start-up incubators) act as intermediaries between academia and industry, guiding technology developers throughout the transition from initial idea conception to market launch. Business support organisations bring together researchers, emerging companies and well-established businesses, aiming to catalyse synergies across the value chain and help secure funding. End-users, including pharmaceutical companies, leverage IVB to streamline research and reduce animal testing. Contract development and manufacturing organisations (CDMOs) ensure IVB scalability for large-scale production and regulatory compliance, while contract research organisations (CROs) offer specialised testing services and regulatory support. Regulatory agencies ensure that IVB-derived products meet stringent safety, efficacy, and quality requirements. The entire IVB ecosystem benefits from both public and private funding, which finances IVB research and development, as well as scholarships and grants to foster academic growth and discoveries. Additionally, consultants offer strategic guidance to optimise IVB development, ensure regulatory compliance with evolving regulatory standards, and enhance market success.

The IVB sector within the EU is struggling with a lack of competitiveness when compared with North American companies (Figure 1C,D). However, there are several factors that could address this. Ongoing efforts from regulatory agencies and governments to phase out animal-based studies^x^ are driving the demand for more human-relevant methods, since adoption of IVB technologies in drug discovery and safety assessment can potentially reduce drug attrition rates. Factors working against this include fragmented research and technology transfer to the market, high implementation costs, limited data sharing, lack of confidence in the technology, and a complex EU regulatory framework that often requires compliance with both EU and national legislation (Figure 2B).

## Going forward

The development and adoption of IVB technologies can transform several sectors, from pharmaceuticals to food safety, strengthening the EU’s competitiveness and resilience. While the EU stands out in academic research [9], translating IVB advancements into industrial settings remains challenging.

Enhancing EU IVB competitiveness requires action at different levels. Dedicated funding, including public–private partnerships and existing initiatives like the EIC (European Innovation Council)xii, could support technology transfer efforts with tailored programs for early-stage developments (e.g., patenting, business model development, professional development training courses for researchers) and later-stage developments (e.g., licencing agreements for technologies with strong business and regulatory potential). CROs, technology developers, and end-user industries could help define investment priorities, while technology transfer offices can mediate between developers and funders. Additionally, funding resources could incentivise the early adoption of standards in IVB technologies to enhance the translational potential, regulatory acceptance, and industrial scalability of IVB products and services [10,11]. Funding calls could explicitly require adherence to relevant standards to ensure interoperability and facilitate earlier market entry.

Promoting data transparency is crucial for increasing confidence in IVB models and methods. Sharing IVB-generated data (e.g., related to pharmacology, metabolism, immunotoxicity) could facilitate broader IVB technology evaluation and adoption. Private companies could be incentivised to share best practices and success stories in IVB for drug development through regulatory benefits like expedited evaluation, fee waivers, or extended market exclusivity for therapeutic compounds. A similar approach could also apply to non-dossier data, supporting the use of safe harbour agreements to promote knowledge sharing without compromising intellectual property rights.

Furthermore, establishing centralised IVB infrastructures within universities and research centres, including core facilities and centres of excellence, could reduce the costs associated with IVB implementation. The integration of highly trained personnel and expensive equipment into specialised facilities would enhance standardisation, improve accessibility, and accelerate market entry for IVB products and services.

Finally, a unified and coordinated EU strategy could simplify regulatory pathways, shorten the time to market, and boost the uptake and use of IVB services and products. The Life Science Strategyxi aims to position the EU as the world’s most attractive place for life sciences and biotechnologies. Moreover, European Agenciesxiii [12,13] could enable faster and more streamlined uptake of IVB methods for regulatory decision-making. By addressing these challenges, the EU can drive IVB innovation and strengthen its global competitiveness. Ultimately this will ensure that EU citizens have access to more efficacious and safer medicines and are better protected from exposure to hazardous chemicals.

## Declaration of interests

All authors are employees of the European Commission’s Joint Research Centre (JRC). The authors declare that they have no commercial, financial, or personal relationships that could be construed as potential conflicts of interest. The views expressed in this short article are solely those of the authors and do not necessarily reflect the official position, policies, or views of the JRC.

## Resources

^i^ https://research-and-innovation.ec.europa.eu/document/download/47554adc-dffc-411b-8cd6-b52417514cb3_en

^ii^ www.oecd.org/en/publications/revised-proposal-for-the-revision-of-the-statistical-definitions-of-biotechnology-and-nanotechnology_085e0151-en.html

^iii^ https://commission.europa.eu/topics/eu-competitiveness/draghi-report_en

^iv^ https://health.ec.europa.eu/medicinal-products/pharmaceutical-strategy-europe_en

^v^ https://environment.ec.europa.eu/strategy/chemicals-strategy_en

^vi^ https://commission.europa.eu/strategy-and-policy/priorities-2019-2024/europe-fit-digital-age/european-industrial-strategy_en

^vii^ https://eur-lex.europa.eu/resource.html?uri=cellar:5bf4e9d0-71d2-11eb-9ac9-01aa75ed71a1.0001.02/DOC_1&format=PDF

^viii^ https://strategic-technologies.europa.eu/index_en

^ix^ https://research-and-innovation.ec.europa.eu/research-area/industrial-research-and-innovation/eu-valorisation-policy_en

^x^ https://single-market-economy.ec.europa.eu/sectors/chemicals/reach/roadmap-towards-phasing-out-animal-testing_en

^xi^ https://research-and-innovation.ec.europa.eu/strategy/strategy-research-and-innovation/jobs-and-economy/strategy-european-life-sciences_en

^xii^ https://eic.ec.europa.eu/index_en

^xiii^ www.ema.europa.eu/en/documents/other/consolidated-3-year-rolling-work-plan-non-clinical-domain-2025-2027_en.pdf
